# Luteolin Inhibits NLRP3 Inflammasome Activation to Ameliorate DSS‐Induced Colitis by Regulating AMPK Signalling

**DOI:** 10.1111/cpr.70134

**Published:** 2025-10-12

**Authors:** Lianxiang Luo, Fangfang Huang, Guixuan Fang, Yiming Sun, Liyan Deng, Yinglin Liao, Xinming Chen, Zhuosi Chen, Xinxun Lin

**Affiliations:** ^1^ School of Ocean and Tropical Medicine Guangdong Medical University Zhanjiang Guangdong China; ^2^ Graduate School Guangdong Medical University Zhanjiang Guangdong China; ^3^ The First Clinical College Guangdong Medical University Zhanjiang Guangdong China

## Abstract

Luteolin alleviates DSS‐induced ulcerative colitis in mice by targeting the NLRP3 inflammasome, as it shows no effect in NLRP3^‐/‐^ mice. It inhibits NLRP3 activation and IL‐1β secretion in macrophages by reducing ROS, mtROS and calcium levels via AMPK binding and signalling. Metabolomic changes suggest lipid metabolism involvement. Luteolin represents a promising NLRP3‐targeted therapeutic candidate for UC.

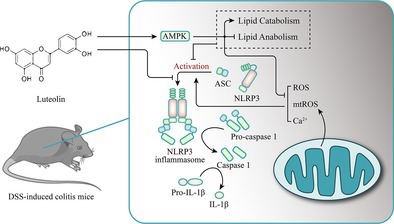

To the Editor,

1

Inflammatory bowel disease (IBD) is a chronic intestinal immune‐inflammatory disease with unknown aetiology, including Crohn's disease (CD) and ulcerative colitis (UC) [1, 2]. Currently, the incidence and prevalence of UC are increasing worldwide, posing a huge challenge to public health [3, 4]. At present, anti‐tumour necrosis factor (anti‐TNF) agents are the first therapeutic choice for the treatment of moderate to severe UC, but they are very costly and may lead to some serious adverse effects [5, 6]. Therefore, there is an urgent need to explore new therapeutic agents for the treatment of UC. Inflammasomes are a group of cytosolic protein complexes whose main function is to recognise various stresses, exogenous microorganisms and endogenous danger signals [7, 8]. Aberrant activation of the NLRP3 inflammasome is closely associated with UC, and currently, the NLRP3 inflammasome has been suggested as a promising therapeutic target for the treatment of UC [9]. Luteolin is a natural dietary flavonoid commonly found in various types of fruits, vegetables and herbs [10]. Luteolin has been reported to have multiple biological activities, including anti‐inflammatory, immune regulation, anti‐infection, antioxidant and anticancer [11]. A small number of studies have proposed that luteolin has the effect of inhibiting NLRP3 inflammasome activation and can alleviate UC [12, 13, 14]. However, the specific mechanism of NLRP3 inflammasome regulation has not been suggested. Our study focuses on revealing the specific pathway mechanism and related metabolism of luteolin in regulating NLRP3 inflammasomes, providing new insights into the potential of NLRP3 inflammasomes as a therapeutic target for UC.

To investigate the effect of luteolin in vivo, we created a colitis model in C57BL/6 mice with 3% DSS in their drinking water for 8 days. The results showed that luteolin could effectively reduce the symptoms of DSS‐induced colitis in WT mice, as evidenced by the decreased weight loss (Figure S1A), DAI score (Figure S1B) and colon shortening (Figure S1C). Histological analysis revealed that luteolin‐treated mice had less inflammation, intact colonic structure and no mucosal damage (Figure S1D,E). Additionally, the colonic MPO activity was significantly lower in luteolin‐treated mice than in the DSS group (Figure S1F). Immunohistochemical staining also showed that the expression of tight junction proteins (including ZO‐1, occludin, claudin‐1 and MUC‐2) was significantly higher in the colon tissue of luteolin‐treated mice, and there were fewer F4/80^+^ macrophage infiltrates at the mucosa of the colonic lesion site (Figure S1D,G). The Western blot analysis of colon tissue also showed that luteolin had the effect of enhancing intestinal barrier protein expression (including ZO‐1, occludin, claudin‐1 and MUC‐2), which was consistent with the results of Immunohistochemical staining (Figure S2). These results indicate that luteolin treatment can effectively alleviate DSS‐induced colitis in mice.

It has been suggested that the NLRP3 inflammasome is a key factor in the development of colitis in mice caused by DSS. To further study the anti‐colitis action of luteolin, we examined the NLRP3 inflammasome in acute colitis. Figure S3 showed that luteolin significantly decreased the levels of cleaved‐caspase‐1 and cleaved‐IL‐1β in mouse colon tissues. We also observed a decrease in IL‐1β levels in mouse colon tissues when treated with luteolin. The reduction of F4/80 macrophages together with the decrease in pro‐inflammatory factors suggests that inflammation may be relieved. In conclusion, these results suggest that luteolin has the potential to ameliorate DSS‐induced colitis by restraining the NLRP3 inflammasome.

To further investigate whether the protective effect of luteolin in colitis depends on the NLRP3 inflammasome, we performed validation in NLRP3^−/−^ mice. As shown in Figure S1, luteolin failed to ameliorate DSS‐induced body weight loss (Figure S1H), DAI score (Figure S1I) and shortening of colon length (Figure S1J) in NLRP3^−/−^ mice. In addition, histological analysis and immunohistochemical staining showed that luteolin treatment also failed to ameliorate crypt destruction, inflammatory cell infiltration, mucosal damage, decreased expression of tight junction proteins and F4/80^+^ macrophage infiltration in the colonic tissues of NLRP3^−/−^ mice (Figure S1K–M). Altogether, these results indicated that the anti‐colitis effect of luteolin was abolished in NLRP3^−/−^ mice.

To further explore the inhibitory effect of luteolin on NLRP3 inflammasomes, we conducted in vitro experiments. We investigated the effect of luteolin on NLRP3 inflammasome activation in vitro by measuring IL‐1β secretion in LPS‐primed THP‐1 cells pre‐treated with luteolin for 30 min before treatment with NLRP3 activators nigericin and ATP. Our results showed that luteolin effectively inhibited caspase‐1 activation and IL‐1β secretion in THP‐1 cells. To further validate our findings, we conducted a similar experiment with primary bone marrow‐derived macrophage (BMDM) cells. Figure S4 revealed that the NLRP3 inflammasome was significantly activated in LPS‐primed BMDM cells following stimulation with nigericin or ATP, and pre‐treatment with luteolin notably reduced nigericin and ATP‐induced NLRP3 inflammasome activation and IL‐1β secretion in BMDM cells. Collectively, these results suggest that luteolin is capable of suppressing NLRP3 inflammasome activation in vitro. The oligomerisation of ASC is a necessary step for NLRP3 inflammasome activation, which in turn triggers caspase‐1 and the release of IL‐1β [15]. To examine the effect of luteolin on ASC oligomerisation, we conducted a chemical cross‐linking assay and immunofluorescence. Results from both THP‐1 cells and BMDM cells showed that luteolin could indeed inhibit ASC oligomerisation, which is consistent with its inhibitory effect on caspase‐1 activation and IL‐1β secretion (Figure 1E,F). It has been hypothesised that Ca^2+^ mobilisation, as well as the production of ROS and mtROS, are responsible for activating the NLRP3 inflammasome [16, 17]. Our analysis of the effect of luteolin on cellular signalling associated with NLRP3 inflammasome activation showed that luteolin pre‐treatment was able to significantly reduce the excessive production of intracellular ROS, mtROS and Ca^2+^ (Figure 1G–I). This indicates that luteolin can inhibit NLRP3 inflammasome assembly by blocking ASC oligomerisation and suppress NLRP3 inflammasome activation by inhibiting ROS, mtROS and Ca^2+^ generation.

**FIGURE 1:**
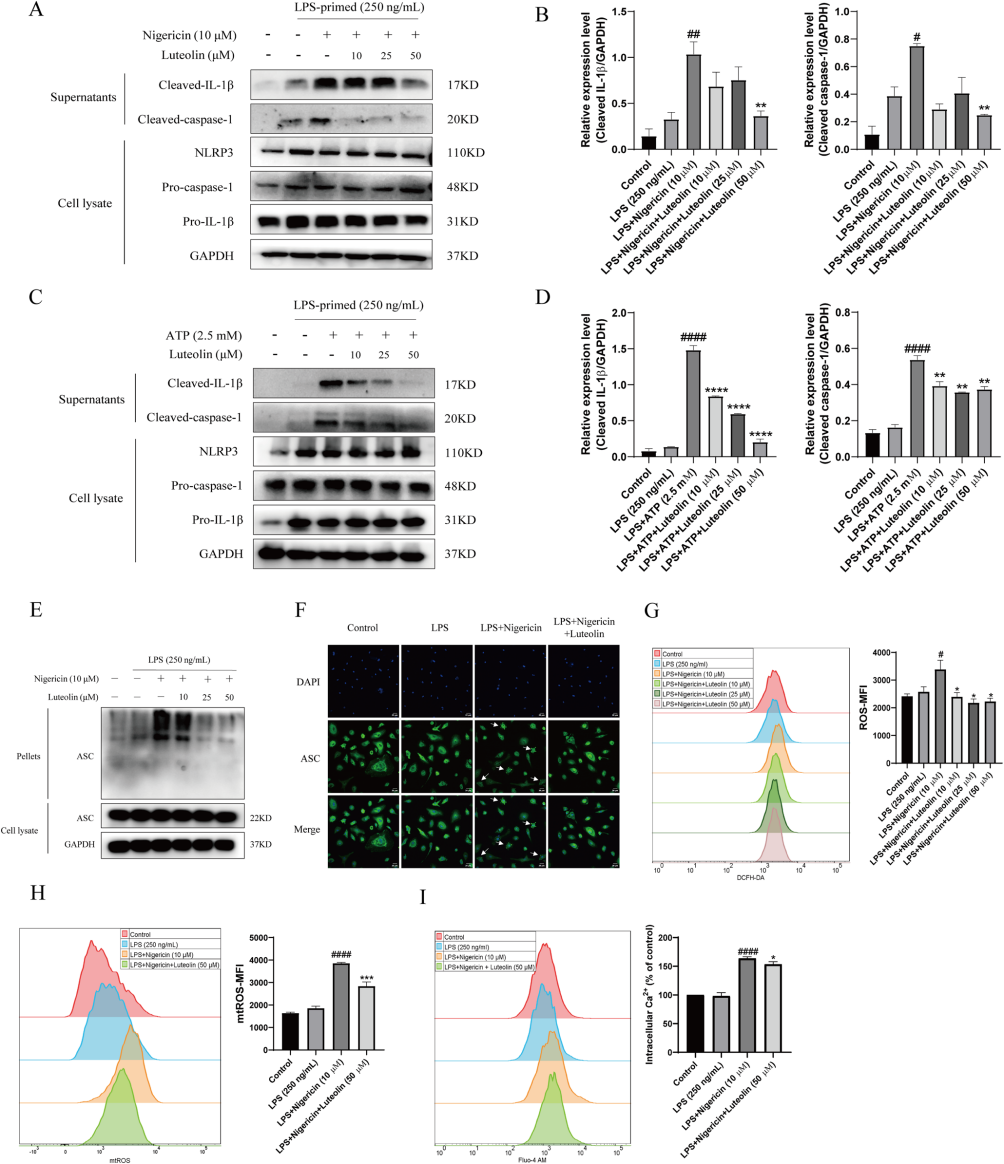
Luteolin inhibited NLRP3 inflammasome activation and reduced ROS, mtROS and Ca^2+^ generation. (A and B) THP‐1 cells were primed with LPS for 4 h, followed by luteolin treatment 30 min before stimulation with nigericin for 30 min. Western blot was used to detect the secretion of cleaved‐caspase‐1 and cleaved‐IL‐1β in the supernatant and the expression of NLRP3, pro‐caspase‐1, pro‐IL‐1β and GAPDH in cell lysate. The grey statistical analysis of Western blot bands was performed by ImageJ software and GraphPad Prism software. Compared to the LPS + Nigericin group, **p < 0.01, ****p < 0.0001. Compared to the LPS group, #*p* < 0.05, ##*p* < 0.01. (C and D) THP‐1 cells were primed with LPS for 4 h, followed by luteolin treatment 30 min before stimulation with ATP for 30 min. Western blot was used to detect the secretion of cleaved‐caspase‐1 and cleaved‐IL‐1β in the supernatant and the expression of NLRP3, pro‐caspase‐1, pro‐IL‐1β and GAPDH in cell lysate. The grey statistical analysis of Western blot bands was performed by ImageJ software and GraphPad Prism software. Compared to the LPS + ATP group, **p < 0.01, ****p < 0.0001. Compared to the LPS group, ####*p* < 0.0001. (E) THP‐1 cells were primed with LPS for 4 h, followed by luteolin treatment 30 min before stimulation with nigericin for 30 min. ASC oligomerisation was detected by a chemical cross‐linking assay. (F) BMDM cells were primed with LPS for 4 h, followed by luteolin treatment 30 min before stimulation with nigericin for 30 min. ASC oligomerisation was detected by immunofluorescence. Scale = 20 μm. (G‐I) THP‐1 cells were primed with LPS for 4 h, followed by luteolin treatment 30 min before stimulation with nigericin for 30 min. Intracellular ROS (G), mtROS (H) and Ca^2+^ (I) production were detected by flow cytometry. Compared to the LPS + Nigericin group, *p < 0.05 and ***p < 0.001. Compared to the LPS group, #*p* < 0.05, ###*p* < 0.001 and ####*p* < 0.0001. Differences were analysed by one‐way ANOVA in GraphPad Prism software. Each experiment was independently performed more than two times.

To further explore the specific mechanism by which luteolin inhibits the activation of NLRP3 inflammasomes, we focused our research on AMPK, a key controller of energy homeostasis. The AMPK signalling pathway is believed to be the primary pathway that controls NLRP3 inflammasome activation [18]. We hypothesised that the AMPK signalling pathway may be involved in the inhibitory effect of luteolin on NLRP3 inflammasome activation. Indeed, luteolin treatment significantly increased the expression of AMPK Thr172‐phosphorylation level compared to LPS plus nigericin treatment (Figure 2A,B). The combination of Compound C (an AMPK inhibitor) can inhibit the activation of AMPK and relieve the inhibition of luteolin on NLRP3 inflammasome (Figure 2C–E). Furthermore, the inhibitory effect of luteolin on intracellular ROS (Figure 2F) and Ca^2+^ (Figure 2G) production was blocked after co‐treatment with Compound C. To further investigate the mechanism, THP‐1 cells were transfected with AMPK siRNA (siAMPK). Western blot results showed that AMPK protein expression was significantly decreased in THP‐1 cells after AMPK siRNA transfection (Figure 2H). As shown in Figure 2I–K, luteolin treatment inhibited caspase‐1 activity, IL‐1β secretion and the accumulation of ROS. However, the effects of luteolin were abolished by siAMPK. In addition, Co‐IP experiments showed enhanced co‐immunoprecipitation signal of luteolin‐treated tagged proteins, suggesting that luteolin has a relationship that promotes the interaction between AMPK and NLRP3. This further illustrates the effect of luteolin in activating AMPK and thus inhibiting NLRP3 (Figure S5). Collectively, these results suggest that luteolin suppresses intracellular ROS and Ca^2+^ production by increasing AMPK phosphorylation, thereby inhibiting NLRP3 inflammasome activation.

**FIGURE 2:**
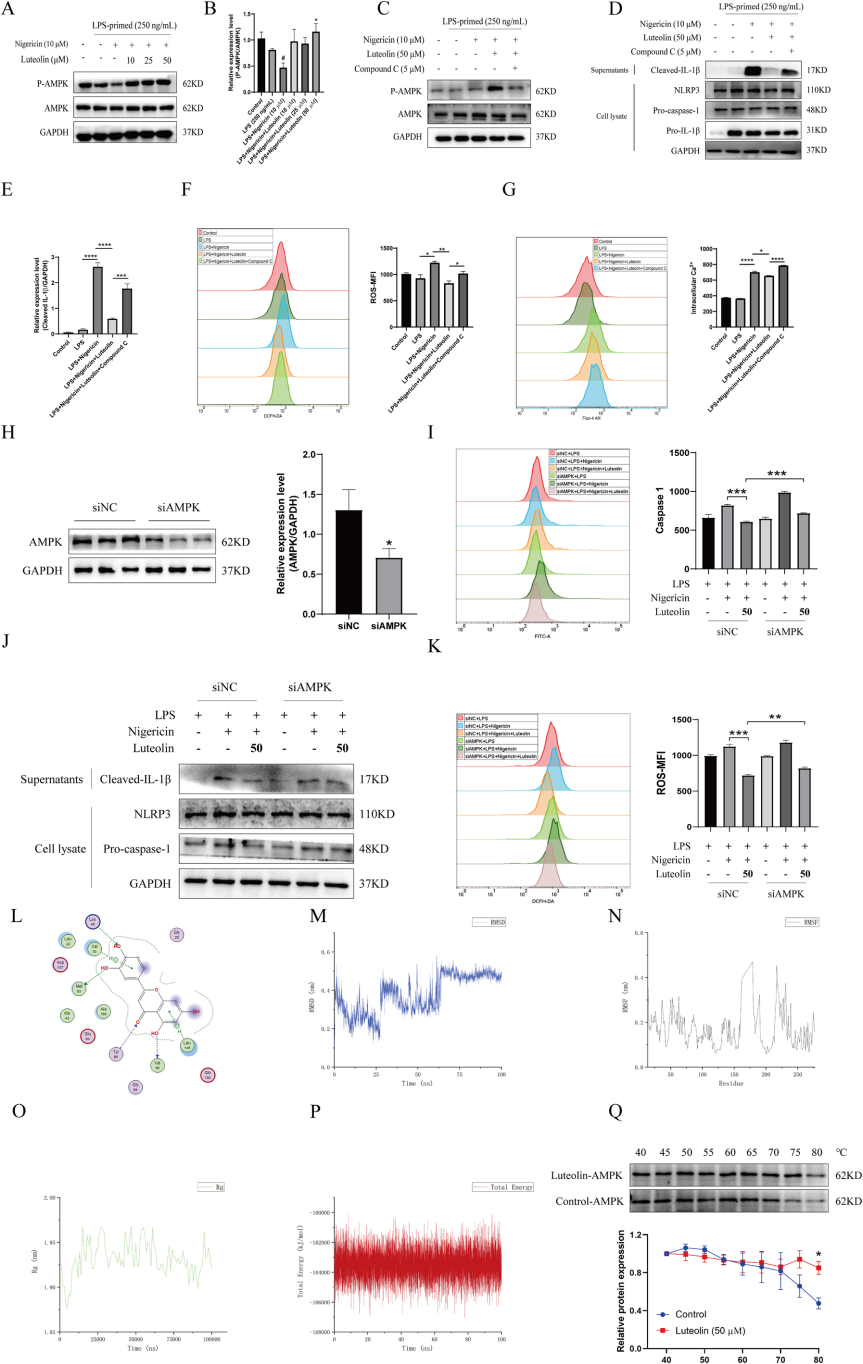
Luteolin activated AMPK by directly binding to the AMPK protein to inhibit the NLRP3 inflammasome. (A and B) THP‐1 cells were primed with LPS for 4 h, followed by luteolin treatment 30 min before stimulation with nigericin for 30 min. Western blot was used to detect the expression of AMPK, P‐AMPK and GAPDH in cell lysate. The grey statistical analysis of Western blot bands was performed by ImageJ software and GraphPad Prism software. Compared to the LPS + nigericin group, *p < 0.05. Compared to the LPS group, #*p* < 0.05. (C–E) THP‐1 cells were primed with LPS for 4 h, followed by luteolin and Compound C treatment 30 min before stimulation with nigericin for 30 min. Western blot was used to detect the secretion of cleaved‐IL‐1β in supernatant and the expression of AMPK, P‐AMPK, NLRP3, pro‐caspase‐1, pro‐IL‐1β and GAPDH in cell lysate. The grey statistical analysis of Western blot bands was performed by ImageJ software and GraphPad Prism software. *p < 0.05, **p < 0.01, ***p < 0.001 and ****p < 0.0001. (F and G) Flow cytometry was used to detect the production of intracellular ROS (F) and Ca^2+^ (G). *p < 0.05 and ****p < 0.0001. (H) Western blot results showed that AMPK was successfully knocked down by siRNA in THP‐1 cells. The grey statistical analysis of Western blot bands was performed by ImageJ software and GraphPad Prism software. Compared to the siNC group, *p < 0.05. (I‐K) After AMPK transfection by siRNA, THP‐1 cells were primed with LPS for 4 h, followed by luteolin (50 μΜ) treatment 30 min before stimulation with nigericin for 30 min. Flow cytometry was used to detect the caspase‐1 activity (I) and the production of intracellular ROS (J). Western blot was used to detect the secretion of cleaved‐IL‐1β in supernatant and the expression of NLRP3, pro‐IL‐1β and GAPDH in cell lysate (K). **p < 0.01 and ***p < 0.001. (L) 2D diagram of the interaction between luteolin and AMPK structure. (M) Change in conformational RMSD of the ligand small molecule luteolin. (N) Change in RMSF of the protein residues. (O) Change in radius of gyration (Rg) of the protein structure. (P) Overall energy change of the complexed system. (Q) Cellular thermal shift assay of AMPK with luteolin. Compared to the control group, *p < 0.05. Differences were analysed by one‐way ANOVA or *t*‐test in GraphPad Prism software. Each experiment was independently performed more than two times.

In addition, we studied the connection between luteolin and AMPK protein. Molecular docking was used to assess luteolin's ability to bind to AMPK. A dock score of less than −5 is usually seen as a sign of successful binding. Figure 2L reveals that luteolin has good binding capacity to AMPK with a dock score of −6.0691. Luteolin interacted with several residues in the protein such as VAL30, LYS45, MET93, TYR95, VAL96 and LEU146. To confirm the stability of the Luteolin‐AMPK bond, molecular dynamics simulation was employed. The RMSD of the ligand in relation to the protein structure gave insight into the steadiness of the binding. The conformational RMSD values of luteolin showed a significant jump at around 29 ns and 65 ns, but eventually reached equilibrium after 65 ns. Most of the RMSD values were within the range of 0.2–0.5 nm, indicating stable binding to the AMPK protein. The RMSF values of the residues involved in the interaction (Val30, Lys45 and Met93‐Val96 region) were consistently lower than 0.3 nm, confirming the stability of the ligand‐protein receptor complex. The radius of gyration (Rg) of the complex was below 1.975 nm, showing that the structure of the complex remained solid throughout the simulation (Figure 2M,N). The global potential energy of the complex was estimated to be −103,489.6604 kJ/mol, further confirming the stable binding of luteolin to AMPK protein (Figure 2O,P). To determine if AMPK is a direct target of luteolin in a cellular context, a cell lysate CETSA experiment was conducted. Results from Figure 2Q showed that luteolin (50 μM) treatment of THP‐1 cells led to a significant thermal stabilisation of AMPK compared to the control group. Thus, these results demonstrate that AMPK is a direct target of luteolin.

Furthermore, studies have increasingly demonstrated that the NLRP3 inflammasome is regulated by variations in intracellular metabolic pathways [19]. In this study, our results showed that luteolin may regulate cellular metabolism through the AMPK signalling pathway. To further explore the molecular mechanism of luteolin's regulation of NLRP3 inflammasome activation, we conducted an untargeted metabolomics analysis of wild‐type mice sera. The OPLS‐DA model (VIP > 1, *p* value < 0.05) identified 25 significantly different metabolites, including glycerophospholipids, fatty acyls, pyridines and derivatives, benzene and substituted derivatives, indoles and derivatives, steroids and steroid derivatives, organooxygen compounds, sphingolipids and others (Figure S6). KEGG pathway enrichment analysis showed that 14 pathways were notably altered (*p* value < 0.05) in the DSS group compared to the control group, the top five being biosynthesis of unsaturated fatty acids, linoleic acid metabolism, cholesterol metabolism, choline metabolism in cancer and bile secretion (Figure S6A). Similarly, five metabolic pathways were significantly different (*p* value < 0.05) in the DSS plus luteolin group in comparison to the DSS group, such as choline metabolism in cancer, steroid hormone biosynthesis, glycerophospholipid metabolism, aldosterone‐regulated sodium reabsorption and biosynthesis of unsaturated fatty acids (Figure S6B). The common differential metabolic pathways among all three groups were choline metabolism in cancer, glycerophospholipid metabolism and biosynthesis of unsaturated fatty acids.

In summary, we aimed to investigate the effect and mechanism of luteolin on NLRP3 inflammasome activation in vitro and in vivo. In this experiment, we utilised a DSS‐induced colitis model to explore the effect of luteolin on the NLRP3 inflammasome in vivo. Our results suggested that luteolin had a positive effect on acute colitis in wild‐type mice, such as reducing weight loss, colon length, inflammatory cell infiltration and decreasing tight junction protein expression. Moreover, luteolin also significantly decreased the IL‐1β expression in the colon tissue of wild‐type mice. However, this anti‐colitis effect of luteolin was not observed in NLRP3^−/−^ mice, implying that the efficacy of luteolin is dependent on NLRP3 expression. To further verify the findings of our in vivo experiments, we conducted in vitro experiments with THP‐1 cells and BMDM cells. It was observed that luteolin could reduce the amount of IL‐1β in the cell culture supernatant and impede NLRP3 inflammasome activation in macrophages. To further explore the effect of luteolin on intracellular ROS, mtROS and Ca^2+^ flux, we conducted experiments and found that luteolin significantly inhibited the excessive production of these molecules. This suggests that luteolin can affect NLRP3 inflammasome assembly and activation in macrophages by regulating the upstream cellular signals. Our research demonstrated that luteolin has the capacity to suppress ROS and Ca^2+^ production by augmenting the phosphorylation of AMPK, thus restraining the activation of the NLRP3 inflammasome. Moreover, our results from molecular docking, molecular dynamics simulation and CETSA experiments showed that AMPK is a direct target of luteolin. We have also preliminarily found that luteolin may be closely related to lipid metabolism in alleviating UC, and further studies will be explored in the future (Figure S7).

## Author Contributions

Lianxiang Luo conceived and designed the study. Fangfang Huang, Guixuan Fang, Yiming Sun, Liyan Deng, Xinming Chen, Zhuosi Chen and Xinxun Lin carried out experiments. Yinglin Liao performed molecular docking. Fangfang Huang, Yiming Sun and Lianxiang Luo wrote the manuscript. Lianxiang Luo reviewed the paper and provided comments. All authors contributed to this manuscript and approved the submitted version.

## Ethics Statement

The Laboratory Animal Welfare and Ethics Committee of Guangdong Medical University approved the animal experiments (GDY2302152), which were performed in accordance with the ‘Guidelines for the Care and Use of Laboratory Animals’.

## Conflicts of Interest

The authors declare no conflicts of interest.

## Supporting information


**Figure S1:** Luteolin relieved DSS‐induced colitis symptoms and colon injury in a NLRP3‐dependent manner. The WT mice and NLRP3^−/−^ mice were randomly divided into three groups (*n* = 8/group): Control groups, DSS groups and DSS + luteolin (50 mg/kg) groups, respectively. (A) Body weight change curve of WT mice in different groups. (B) DAI score of WT mice in different groups. (C) Colon appearance and colon length of WT mice in different groups. (D) Representative pictures of HE staining and immunohistochemical staining of WT mice colon tissues. Image captured at ×20 magnification, scale = 100 μm. (E) Histopathological score of WT mice. (F) MPO activity in colon tissues of WT mice. (G) Percentage of positive area of IHC staining for ZO‐1, occludin, claudin‐1 and F4/80 proteins in WT mice. (H) Body weight change curve of NLRP3^−/−^ mice in different groups. (I) DAI score of NLRP3^−/−^ mice in different groups. (J) Colon appearance and colon length of NLRP3^−/−^ mice in different groups. (K) Representative pictures of HE staining and immunohistochemical staining of NLRP3^−/−^ mice colon tissues. Image captured at ×20 magnification, scale = 100 μm. (L) Histopathological score. (M) Percentage of positive area of IHC staining for ZO‐1, occludin, claudin‐1 and F4/80 proteins in NLRP3^−/−^ mice. The positive staining area was analysed using GraphPad Prism software and ImageJ software. Compared to the Control group, *p < 0.05, **p < 0.01, ***p < 0.001, ****p < 0.0001. Compared to the DSS group, #*p* < 0.05, ##*p* < 0.01, ###*p* < 0.001, ####*p* < 0.0001. Differences were analysed by *t*‐test or one‐way ANOVA in GraphPad Prism software. Each experiment was independently performed more than two times.
**Figure S2:** Luteolin improves the intestinal barrier in intestinal tissues. (A) The Western blot assay was used to detect the expression level of intestinal barrier protein in intestinal tissue (ZO‐1, claudin‐1, occludin and MUC‐2). (B) Western blot bands were analysed by ImageJ software and GraphPad Prism software. Compared to the DSS group, *p < 0.05, **p < 0.01. Differences were analysed by *t*‐test in GraphPad Prism software. Each experiment was independently performed more than two times.
**Figure S3:** Luteolin inhibited caspase‐1 activation and IL‐1β maturation in colon tissues. (A) Western blot was used to detect the expression levels of cleaved‐caspase‐1 and cleaved‐IL‐1β in colon tissues. (B) ELISA was used to detect the levels of IL‐1β in colon tissues of mice in different groups. Compared to the Control group, **p < 0.01. Compared to the DSS group, #*p* < 0.05. Differences were analysed by one‐way ANOVA in GraphPad Prism software. Each experiment was independently performed more than two times.
**Figure S4:** Luteolin inhibited NLRP3 inflammasome activation in BMDM cells. BMDM cells were primed with LPS for 4 h, followed by luteolin treatment 30 min before stimulation with nigericin or ATP for 30 min. (A) The secretion of IL‐1β in supernatants was detected by ELISA. (B) Western blot was used to detect the secretion of cleaved‐caspase‐1 and cleaved‐IL‐1β in supernatant and the expression of NLRP3, pro‐caspase‐1, pro‐IL‐1β and GAPDH in cell lysate. **p < 0.01, ****p < 0.0001. Differences were analysed by one‐way ANOVA in GraphPad Prism software. Each experiment was independently performed more than two times.
**Figure S5:** Luteolin activated AMPK by directly binding to AMPK protein. Co‐immunoprecipitation assays of AMPK and NLRP3 in THP‐1 cells were analysed quantitatively. **p < 0.01. Differences were analysed by *t*‐test in GraphPad Prism software. Each experiment was independently performed more than two times.
**Figure S6:** KEGG pathway enrichment analysis among three groups. (A) DSS group vs. control group. (B) DSS plus luteolin group vs. DSS group.
**Figure S7:** Histogram of significantly different metabolites in three groups: control, DSS and DSS plus luteolin group. Using GraphPad Prism software to analyse data. Differences were analysed by one‐way ANOVA in GraphPad Prism software. Compared to the DSS group, *p < 0.05, **p < 0.01. Compared to the Control group, #*p* < 0.05, ##*p* < 0.01, ####*p* < 0.0001.

## Data Availability

The data that support the findings of this study are available from the corresponding author upon reasonable request.
