# Supplementary material for: Luteolin Inhibits NLRP3 Inflammasome Activation to Ameliorate DSS‐Induced Colitis by Regulating AMPK Signalling
Source: Cell Prolif. 2025 Oct 12;59(2):e70134. doi: 10.1111/cpr.70134 (PMC12877959; doi:10.1111/cpr.70134)
Supplement: Supplementary file 1 — Figure S1: Luteolin relieved DSS‐induced colitis symptoms and colon injury in a NLRP3‐dependent manner. The WT mice and NLRP3−/− mice were randomly divided into three groups (n = 8/group): Control groups, DSS groups and DSS + luteolin (50 mg/kg) groups, respectively. (A) Body weight change curve of WT mice in different groups. (B) DAI score of WT mice in different groups. (C) Colon appearance and colon length of WT mice in different groups. (D) Representative pictures of HE staining and immunohistochemical staining of WT mice colon tissues. Image captured at ×20 magnification, scale = 100 μm. (E) Histopathological score of WT mice. (F) MPO activity in colon tissues of WT mice. (G) Percentage of positive area of IHC staining for ZO‐1, occludin, claudin‐1 and F4/80 proteins in WT mice. (H) Body weight change curve of NLRP3−/− mice in different groups. (I) DAI score of NLRP3−/− mice in different groups. (J) Colon appearance and colon length of NLRP3−/− mice in different groups. (K) Representative pictures of HE staining and immunohistochemical staining of NLRP3−/− mice colon tissues. Image captured at ×20 magnification, scale = 100 μm. (L) Histopathological score. (M) Percentage of positive area of IHC staining for ZO‐1, occludin, claudin‐1 and F4/80 proteins in NLRP3−/− mice. The positive staining area was analysed using GraphPad Prism software and ImageJ software. Compared to the Control group, *p < 0.05, **p < 0.01, ***p < 0.001, ****p < 0.0001. Compared to the DSS group, #p < 0.05, ##p < 0.01, ###p < 0.001, ####p < 0.0001. Differences were analysed by t‐test or one‐way ANOVA in GraphPad Prism software. Each experiment was independently performed more than two times. Figure S2: Luteolin improves the intestinal barrier in intestinal tissues. (A) The Western blot assay was used to detect the expression level of intestinal barrier protein in intestinal tissue (ZO‐1, claudin‐1, occludin and MUC‐2). (B) Western blot bands were analysed by ImageJ software and GraphPad Pri [file CPR-59-e70134-s001.docx]

Supplementary Material

**Supplementary content**

**Luteolin ameliorated DSS-induced colitis by inhibiting the NLRP3 inflammasome through regulating lipid metabolism**

Studies have increasingly demonstrated that the NLRP3 inflammasome is regulated by variations in intracellular metabolic pathways. In this study, our results showed that luteolin may regulate cellular metabolism through the AMPK signaling pathway. To further explore the molecular mechanism of luteolin's regulation of NLRP3 inflammasome activation, we conducted an untargeted metabolomics analysis of wild-type mice sera. The OPLS-DA model (VIP > 1, P-value < 0.05) identified 25 significantly different metabolites, including glycerophospholipids, fatty acyls, pyridines and derivatives, benzene and substituted derivatives, indoles and derivatives, steroids and steroid derivatives, organooxygen compounds, sphingolipids, and others (Supplementary figure 7). KEGG pathway enrichment analysis showed that 14 pathways were notably altered (P-value < 0.05) in the DSS group compared to the control group, the top 5 being biosynthesis of unsaturated fatty acids, linoleic acid metabolism, cholesterol metabolism, choline metabolism in cancer, and bile secretion (Supplementary figure 6A). Similarly, 5 metabolic pathways were significantly different (P-value < 0.05) in the DSS plus luteolin group in comparison to the DSS group, such as choline metabolism in cancer, steroid hormone biosynthesis, glycerophospholipid metabolism, aldosterone-regulated sodium reabsorption, and biosynthesis of unsaturated fatty acids (Supplementary figure 6B). The common differential metabolic pathways among all three groups were choline metabolism in cancer, glycerophospholipid metabolism, and biosynthesis of unsaturated fatty acids.

**Supplementary Figure**

**Supplementary Figure 1**

**
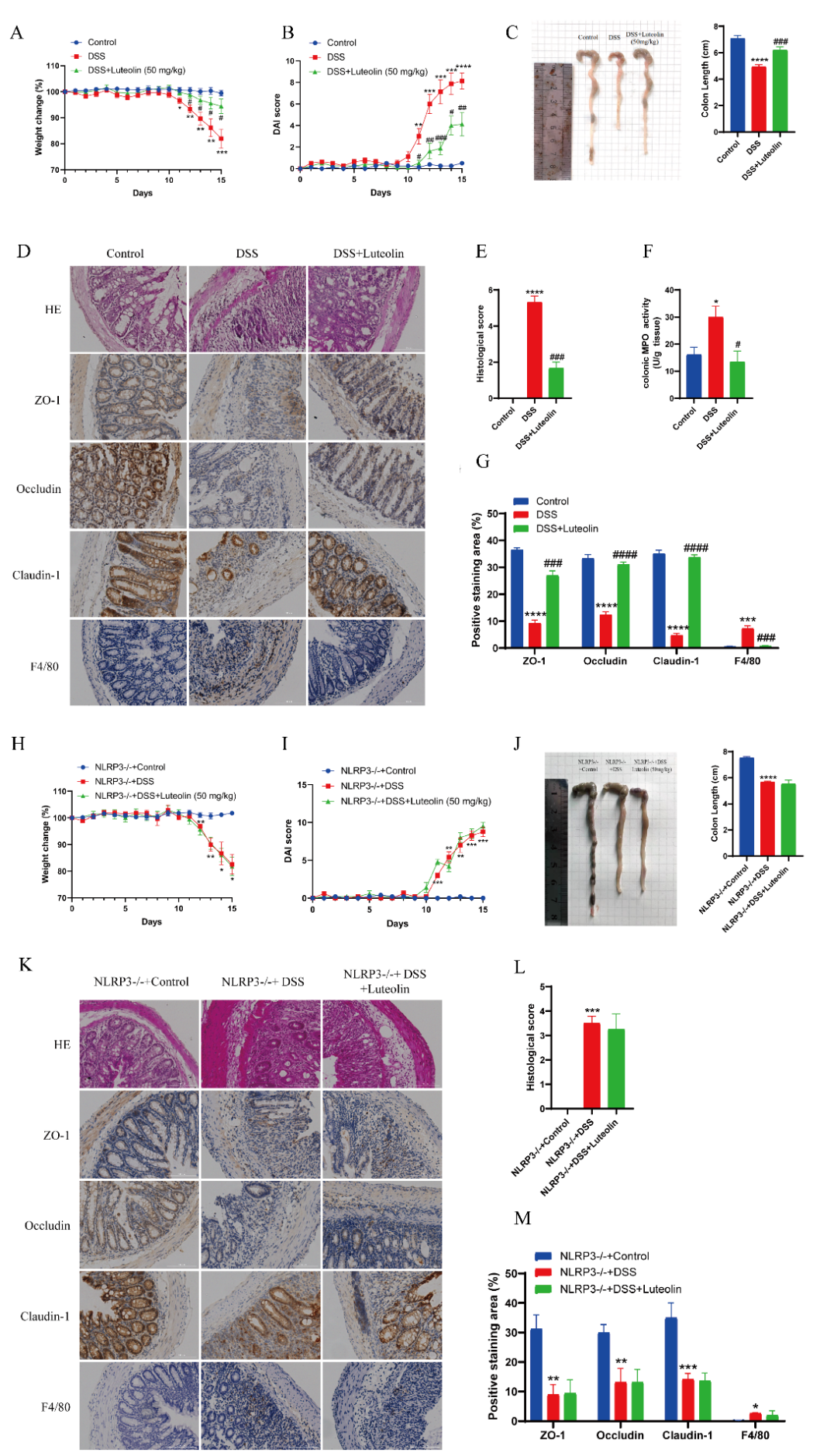
**

**Supplementary figure 1 Luteolin relieved DSS-induced colitis symptoms and colon injury in a NLRP3-dependent manner.** The WT mice and NLRP3 ^-/-^ mice were randomly divided into three groups respectively. (n = 8 /group): Control groups, DSS groups, and DSS + luteolin (50 mg/kg) groups. **(**A) Body weight change curve of WT mice in different groups. **(**B) DAI score of WT mice in different groups. Image captured at×200 magnification, scale＝100μm. **(**C) Colon appearance and colon length of WT mice in different groups. (D) Representative pictures of HE staining and immunohistochemical staining of WT mice colon tissues. Image captured at×20 magnification, scale＝100μm. (E) Histopathological score of WT mice. (F) MPO activity in colon tissues of WT mice. (G) Percentage of positive area of IHC staining for ZO-1, occludin, claudin-1, and F4/80 proteins in WT mice. (H) Body weight change curve of NLRP3 ^-/-^ mice in different groups. (I) DAI score of NLRP3 ^-/-^ mice in different groups. (J) Colon appearance and colon length of NLRP3 ^-/-^ mice in different groups. (K) Representative pictures of HE staining and immunohistochemical staining of NLRP3 ^-/-^ mice colon tissues. Image captured at×20 magnification, scale＝100μm. (L) Histopathological score. (M) Percentage of positive area of IHC staining for ZO-1, occludin, claudin-1, and F4/80 proteins in NLRP3 ^-/-^ mice. The positive staining area were analyzed using Graphpad prism software and ImageJ software. Compared with the control group, **P*<0.05, ***P*<0.01, ****P*<0.001, *****P*<0.0001. Compared with the DSS group, #*P*<0.05, ##*P*<0.01, ###*P*<0.001, ####*P*<0.0001. Differences were analyzed by t-test or one-way ANOVA in Graphpad prism software. Each experiment was independently performed more than twice.

**Supplementary Figure 2**

**
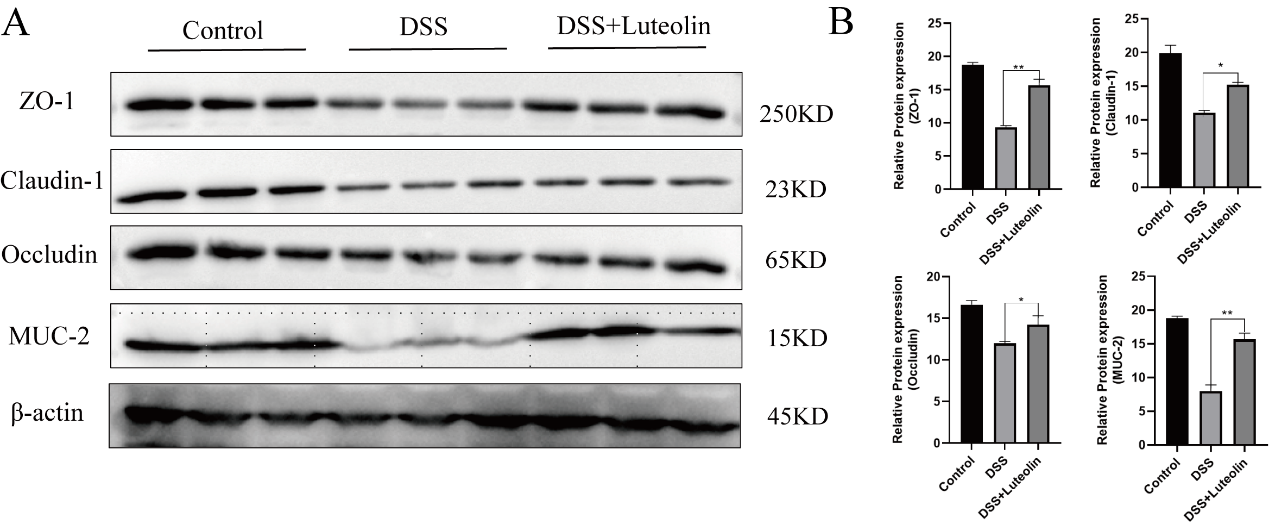
**

**Supplementary figure 2 Luteolin improves the intestinal barrier in intestinal tissues.** (A) The Western bolt assay was used to detect the expression level of intestinal barrier protein in intestinal tissue (ZO-1, Claudin-1, Occludin, MUC-2). (B) Western Blot bands was performed by ImageJ software and Graphpad prism software. Compared with the DSS group, **P*<0.05, ***P*<0.01. Differences were analyzed by t-test in Graphpad prism software. Each experiment was independently performed more than twice.

**Supplementary Figure 3**


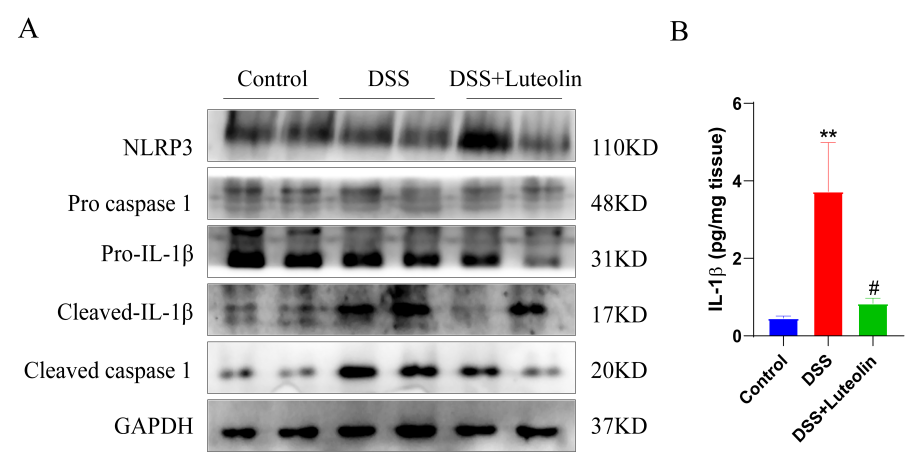


**Supplementary figure 3 Luteolin inhibited caspase-1 activation and IL-1β maturation in colon tissues.** (A) Western blot was used to detect the expression levels of cleaved-caspase-1 and cleaved-IL-1β in colon tissues. (B) ELISA was used to detect the levels of IL-1β in colon tissues of mice in different groups. Compared with the Control group, ***P*<0.01. Compared with the DSS group, #*P*<0.05. Differences were analyzed by one-way ANOVA in Graphpad prism software. Each experiment was independently performed more than twice.

**Supplementary Figure 4**


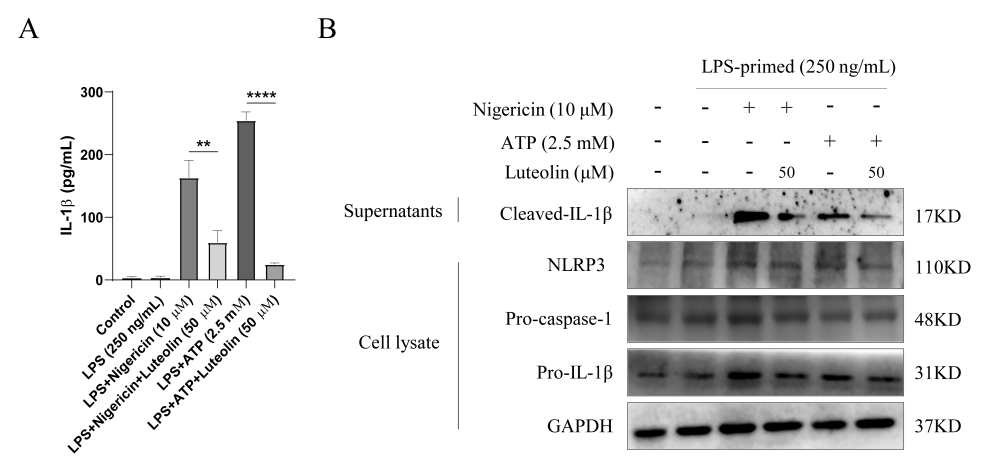


**Supplementary figure 4 Luteolin inhibited NLRP3 inflammasome activation in BMDM cells.** BMDM cells were primed with LPS for 4 h, followed by luteolin treatment 30 min before stimulation with nigericin or ATP for 30 min. (A) The secretion of IL-1β in supernatants was detected by ELISA. (B) Western blot was used to detect the secretion of cleaved-caspase-1 and cleaved-IL-1β in supernatant and the expression of NLRP3, pro-caspase-1, pro-IL‑1β, and GAPDH in cell lysate. **P<0.01, ****P<0.0001. Differences were analyzed by one-way ANOVA in Graphpad prism software. Each experiment was independently performed more than twice.

**Supplementary Figure 5**

**
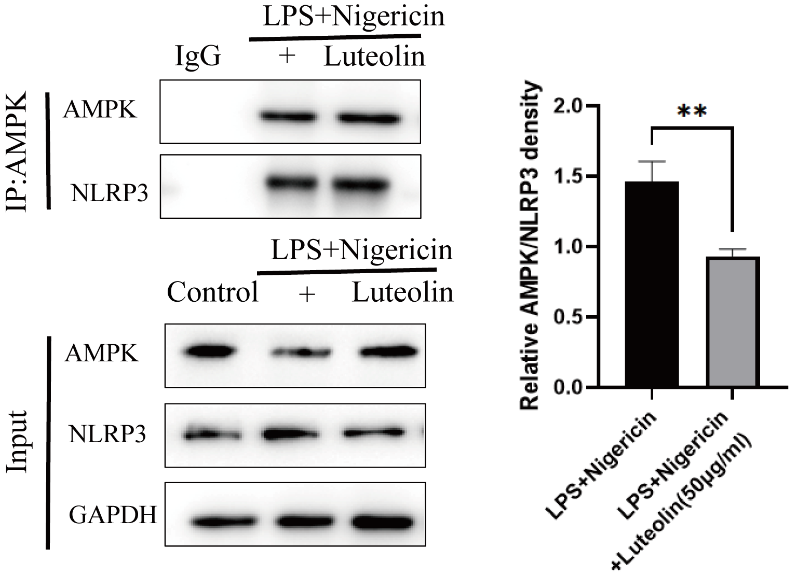
**

**Supplementary figure 5 Luteolin activated AMPK by directly binding to AMPK protein.** Co-immunoprecipitation assays of AMPK and NLRP3 in THP-1 cells and analyzed quantitatively. ***P* < 0.01. Differences were analyzed by t-test in Graphpad prism software. Each experiment was independently performed more than twice.

**Supplementary Figure 6**


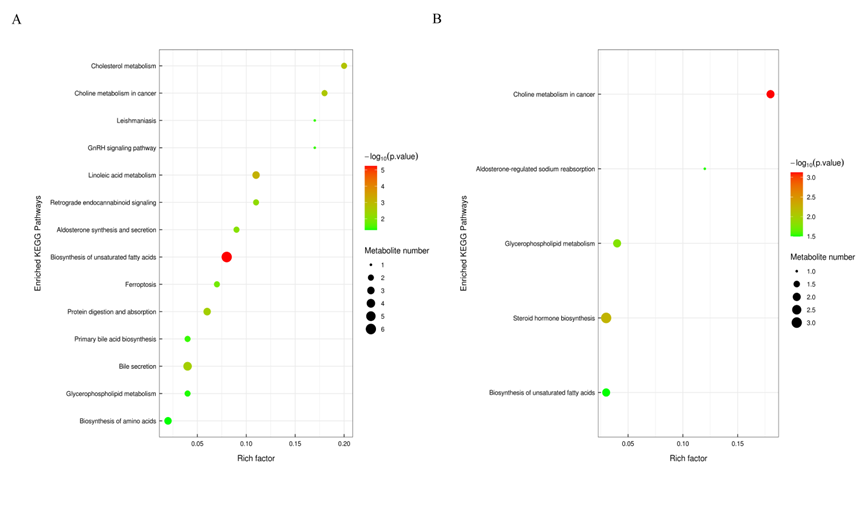


**Supplementary figure 6 KEGG pathway enrichment analysis among three groups.** (A) DSS group vs control group. (B) DSS plus luteolin group vs DSS group.

**Supplementary Figure 7**

**
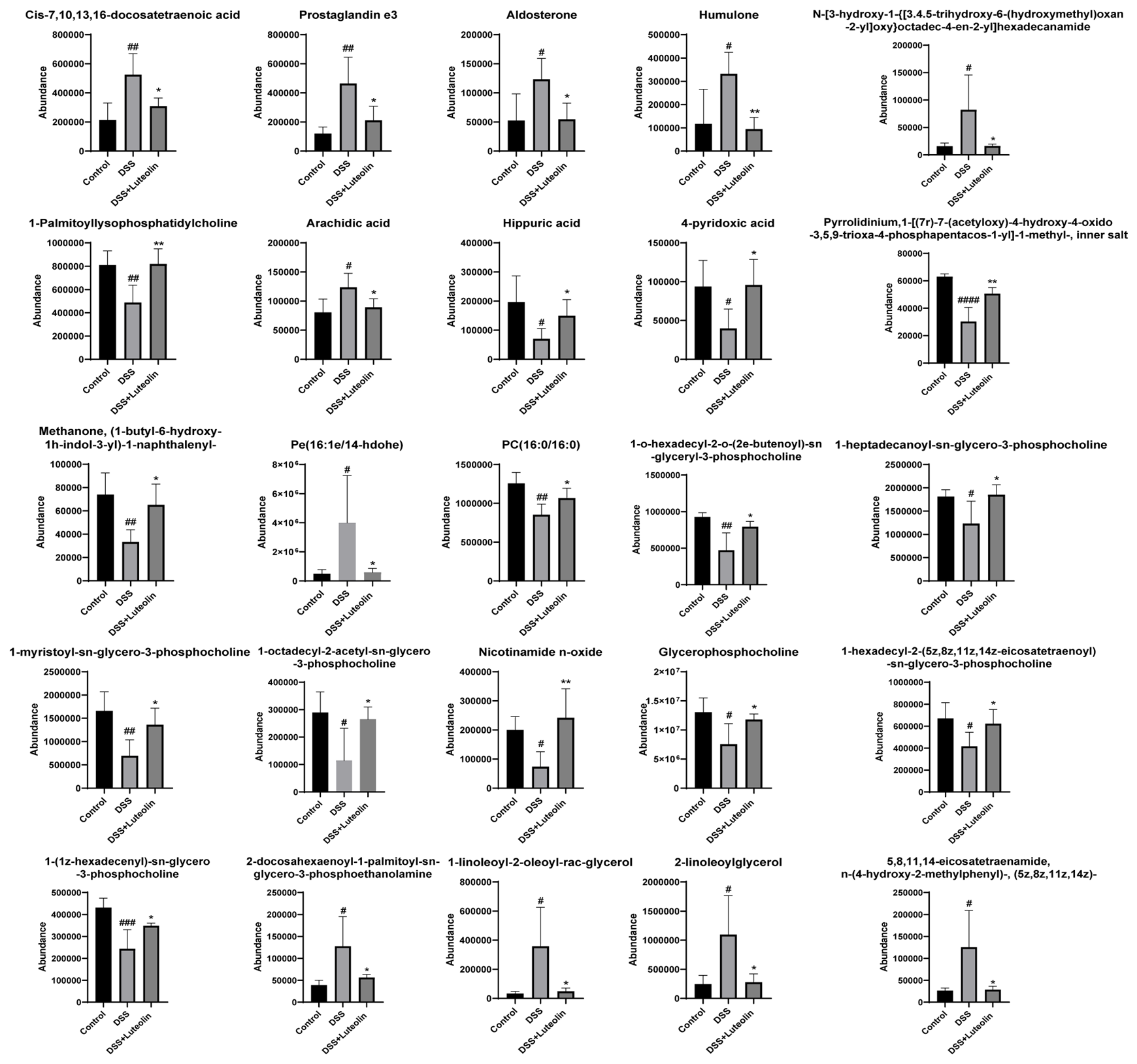
**

**Supplementary figure 7 Histogram of significantly different metabolites in three groups: control, DSS, and DSS plus luteolin group.** Using Graphpad prism software to analyze data. Differences were analyzed by one-way ANOVA in Graphpad prism software. Compared with the DSS group, **P*<0.05, ***P*<0.01. Compared with the Control group, #*P*<0.05, ##*P*<0.01, ####*P*<0.0001.
